# Genomic patient information management system

**DOI:** 10.1186/gb-2010-11-s1-p44

**Published:** 2010-11-04

**Authors:** D Dharmiah, M Narasinga Rao, Ravikiran, N Sandeep, V DS Gopal, Allam Appa Rao, GR Sridhar

**Affiliations:** 1 Department of Computer Sciences, Vignan’s Institute of Information Technology, Visakhapatnam 530046, India and JNTU, Kakinada 533003, India; 2 JNTU, Kakinada 533003, India; 3 Endocrine and Diabetes Centre, 15-12-15 Krishnanagar, Visakhapatnam 530 002, India

## Background

Post-human-genomic-sequencing, efforts are being made to merge data from clinical phenotypes (clinical and biochemical values) with genomic data to provide personalized advice and to improve the efficiency of health care systems. The currently available models require integration, while at the same time ensuring security and stability.

## Components

We developed a model retrieval “Genomic patient information management system” It caters to the requirements of clinicians and patients by acting as an interface which allows 1) Maintaining the patient records with diagnosis reports 2) Sending the next appointment dates to the patients through an SMS facility. 3) E-mail facility to provide interaction b/n patient and doctor. 4) Creating a Semantic network model for storing the genomic information 5) Indexing facility to check the database of genomic information 6) A Search Engine, to search for a key word in the genomic database 7) Offering provable protection from individual re identification based on clinical features 8) Allowing sensitive patterns of genomic codes to be automatically extracted 9) generating anonymization to ensure individual privacy 10) performing clinical case analysis tasks.

## Conclusion

The patient information management application has an intuitive user interface and is flexible for further upgradation
